# Population typing of the causal agent of cassava bacterial blight in the Eastern Plains of Colombia using two types of molecular markers

**DOI:** 10.1186/1471-2180-14-161

**Published:** 2014-06-19

**Authors:** César A Trujillo, Nathalia Arias-Rojas, Lucie Poulin, César A Medina, Anibal Tapiero, Silvia Restrepo, Ralf Koebnik, Adriana J Bernal

**Affiliations:** 1 Laboratorio de Micología y Fitopatología Uniandes (LAMFU), Universidad de Los Andes, Bogotá, Colombia; 2 Institut de Recherche pour le Développement (IRD), UMR Résistance des Plantes aux Bioaggresseurs, Montpellier, France; 3 Corporación Colombiana de Investigación Agropecuaria (CORPOICA), La Libertad, Villavicencio, Colombia

**Keywords:** *Xanthomonas axonopodis* pv. *manihotis*, Population dynamics, Molecular markers, Plant pathology

## Abstract

**Background:**

Molecular typing of pathogen populations is an important tool for the development of effective strategies for disease control. Diverse molecular markers have been used to characterize populations of *Xanthomonas axonopodis* pv. *manihotis* (*Xam*), the main bacterial pathogen of cassava. Recently, diversity and population dynamics of *Xam* in the Colombian Caribbean coast were estimated using AFLPs, where populations were found to be dynamic, diverse and with haplotypes unstable across time. Aiming to examine the current state of pathogen populations located in the Colombian Eastern Plains, we also used AFLP markers and we evaluated the usefulness of Variable Number Tandem Repeats (VNTRs) as new molecular markers for the study of *Xam* populations.

**Results:**

The population analyses showed that AFLP and VNTR provide a detailed and congruent description of *Xam* populations from the Colombian Eastern Plains. These two typing strategies clearly separated strains from the Colombian Eastern Plains into distinct populations probably because of geographical distance. Although the majority of analyses were congruent between typing markers, fewer VNTRs were needed to detect a higher number of genetic populations of the pathogen as well as a higher genetic flow among sampled locations than those detected by AFLPs.

**Conclusions:**

This study shows the advantages of VNTRs over AFLPs in the surveillance of pathogen populations and suggests the implementation of VNTRs in studies that involve large numbers of *Xam* isolates in order to obtain a more detailed overview of the pathogen to improve the strategies for disease control.

## Background

In order to generate effective mechanisms for the control of plant diseases, it is crucial to gain insights into the diversity and population dynamics of plant pathogens [1,2]. Pathogens showing a high genotypic diversity are regarded as being harder to control, because plant resistance can be overcome by more suitable pathotypes [3]. Hence, the development of durable resistance becomes more challenging with this kind of pathogens. Factors such as the genetic flow between pathogen populations and processes that increase the genetic changes of these populations may contribute to break the resistance in monocultures [3-5].

*Xanthomonas axonopodis* pv. *manihotis* (*Xam*) is the causal agent of cassava bacterial blight disease (CBB), the most important bacterial disease of cassava. The most common symptoms of CBB are angular leaf spots, stem exudates, cankers, blight, wilt and dieback [6,7]. *Xam* is an example of a pathogen that presents diverse degrees of variability in different geographical zones and interesting population processes, including genetic flow and instability of populations in different geographical regions [7-10]. *Xam* populations have been characterized in different countries in South America and Africa, starting in the 1980s. These studies showed that the South American populations were more diverse than those from Africa [9,11-14]. Particularly, *Xam* populations from Colombia were classified as highly diverse and showed significant levels of genetic flow between them, in spite of their distant geographical origins in the country [8,9,14]. In the 1990s, *Xam* populations were mainly studied in three regions of Colombia: the Caribbean region, the Eastern Plains and the province of Cauca [8,9,14]. These studies showed that *Xam* populations from the Caribbean and Eastern Plains were dynamic and presented a higher genetic diversity when compared with populations from Cauca [8,9,14]. Recently, we monitored populations of the pathogen in the Caribbean region, where three cassava varieties are intensively and extensively cultivated. These studies were performed using AFLPs and sequences of genes coding for Type Three Effectors proteins (T3Es). In the Caribbean, we commonly found a lack of genetic differentiation among the sampled locations, as a result of potential genotype flow promoted by the exchange of propagative material infected with *Xam*. Additionally, we identified that Caribbean populations change rapidly over time, since it was already possible to establish a temporal differentiation compared to the populations characterized by Restrepo and collaborators in the 1990s [8,15]. Despite the relevance of a constant monitoring of pathogen populations, only those from the Caribbean have being recently studied [15]. However, it is pertinent to characterize populations outside of the studied regions and to establish their dynamics and to which extent those dynamics may have an impact on the crop.

A number of different molecular markers have been implemented for *Xam* population studies. These include Restriction Fragment Length polymorphisms (RFLPs), Enterobacterial Repetitive Intergenic Consensus-PCR (ERIC-PCR) and Amplified Fragment Length Polymorphisms (AFLPs) [12,14,16]. Nevertheless, the most useful markers for population typing of this pathogen are AFLPs [8,10,16]. This is due to their high discriminatory power, when compared to other types of markers previously used, such as RFLPs [16]. However, traditional AFLPs are a time-consuming technique. In addition, it is difficult to standardize the protocols between laboratories because band patterns are not easily coded and the process can become subjective [17,18]. Recently, other typing techniques have been developed to reduce the standardization time, as well as to reduce the time and cost required to obtain the results [17,19]. One of these techniques is based on the sequencing of Variable Number Tandem Repeat (VNTR) loci, which detect polymorphisms in tandem repeats in a given genome and have been important to obtain informative markers [20,21]. VNTRs were implemented more than a decade ago to characterize highly monomorphic human and animal pathogens such as *Mycobacterium tuberculosis*[22,23], *Bacillus anthracis*[24] and *Staphylococcus aureus*[25]. More recently, VNTRs have been implemented to analyze the population genetics and diversity of plant pathogens such as *Xylella fastidiosa*[26], *Xanthomonas citri* pv. *citri*[27], *Ralstonia solanacearum*[28], and the bacterial rice pathogen *Xanthomonas oryzae* pv. oryzicola [29]. VNTRs have allowed to uncover variability that was not detected using other molecular markers [30,31]. An additional advantage of VNTRs compared to other typing techniques is the reduction in costs, which is given by the following factors: first of all, a DNA extraction procedure is often not required because VNTRs can be easily amplified from bacterial colonies. Secondly, the amplification and detection does not require specialized equipment and reagents [21]. Finally, the reduction in the sequencing cost allows the analyses of a higher number of loci and samples, with at a reasonably low cost [17,19]. All these advantages make VNTRs promising molecular markers to study populations of *Xam* when cost is a limiting factor and when the access to especialized laboratory equipment is restricted.

The aim of this study was to evaluate the diversity of current *Xam* populations in the Eastern Plains of Colombia using two types of neutral molecular markers. The Eastern Plains is the second most important region for cassava cultivation in Colombia. In contrast to the Caribbean cassava fields, Eastern Plains fields are considerably small and their growers are not commercially allied for trading of their produce. In this study, we isolated strains from cassava fields located at the provinces of Meta and Casanare, located at the Eastern Plains of Colombia, from 2011 to 2012. The collected isolates were typed using both AFLPs and VNTRs markers. This study highlights the usefulness of VNTR markers for characterizing populations of *Xam*. This study provides an updated distribution of distinct populations of *Xam* in the Eastern Plains of Colombia.

## Methods

### Sampling and bacterial isolation

**Figure 1:**
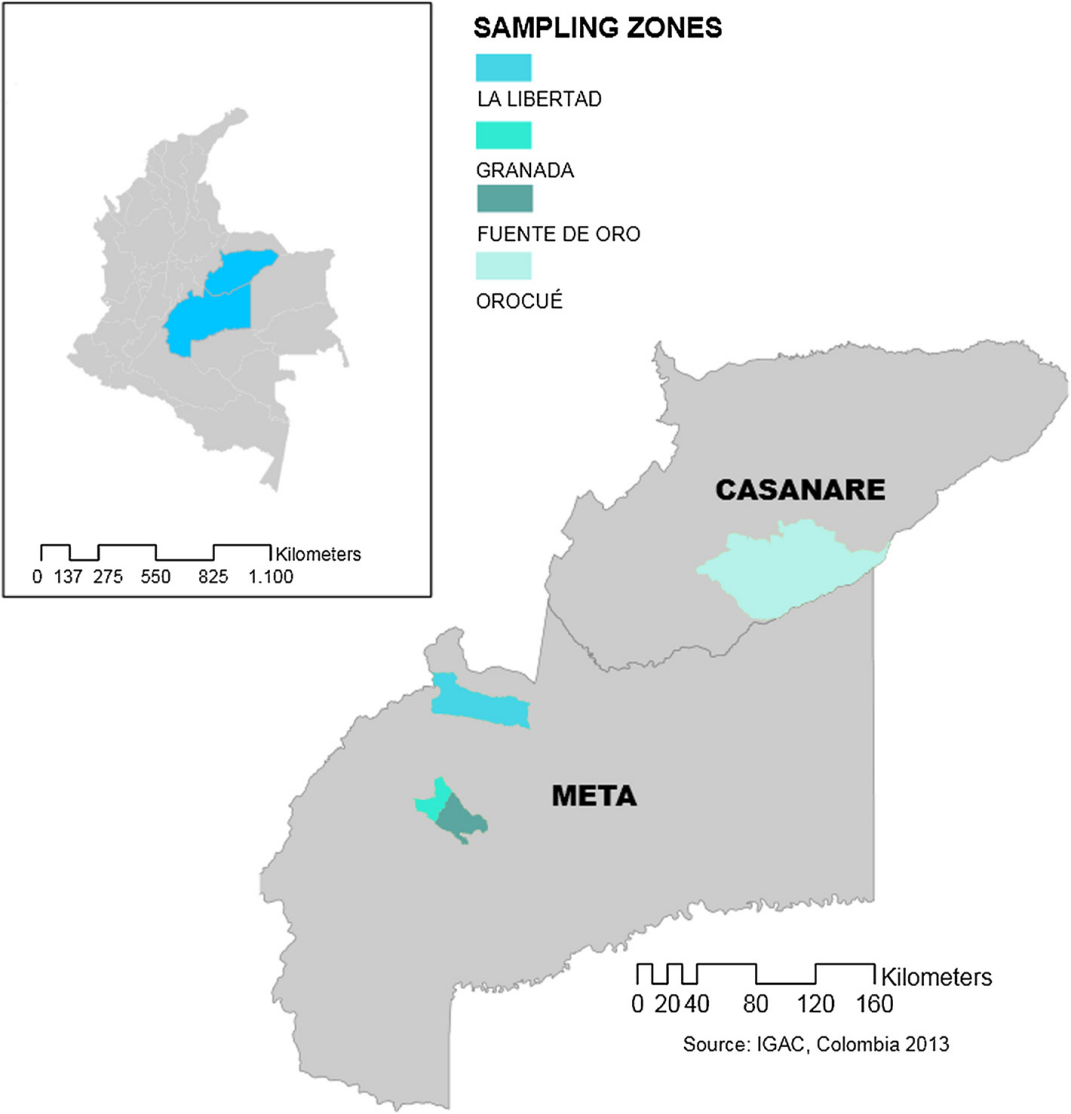
**Sampling zones in the Eastern Plains of Colombia.** Sampling zones were selected between provinces of Meta and Casanare. Shaded areas in the map represent sampled locations.

Cassava crops in the Meta and Casanare provinces of Colombia were sampled from 2011 to 2012 (Figure 1). In Meta, local fields at La Libertad, Granada and Fuente de Oro were visited during 2011. In Casanare, fields near Orocué were sampled in 2012. Sampling was conducted in diagonal transects in three to four fields in each location. Leaves with characteristic CBB symptoms were collected for bacterial isolation. The number of collected samples was dependent on the disease incidence in each field.

For isolation of the bacterium, a 1 cm-diameter leaf disk with infected and healthy tissue was obtained from each sample. The disk was disinfected with 1% hypochlorite and washed in sterile water three times. The tissue was ground in 200 μl of 10 mM MgCl_2_ and two 1:10 serial dilutions were performed. A total of 100 μl of each dilution were plated onto LPGA medium (5 g yeast extract, 5 g dextrose, 5 g Peptone and 15 g agar were used per liter of distilled water) and then incubated at 28°C for 48 h. White, viscous bacterial colonies, typical of *Xam* were found in high populations in all plates coming from symptomatic tissue. These were confirmed as *Xam* using primers directed to the C-terminus of the gene coding for PthB, now called TALE1_*Xam*_[32] (Additional file 1), which is located in the plasmid p44. This region is widely distributed in *Xam* strains and it has been implemented for *Xam* identification [33]. A single colony from each sample was selected to be preserved in 30% glycerol at -80°C. In addition, ten *Xam* strains, which represented the genetic diversity of the pathogen in the 1990s in the Colombian Eastern Plains, were used as reference strains. Reference strains were kindly provided by Dr. Valérie Verdier from IRD (Institut de recherche pour le développement, Montpellier, France).

### DNA extraction and amplification

*Xam* isolates were grown overnight in 5 ml of liquid Phi (Φ) medium (10 g yeast extract, 5 g dextrose and 5 g Casaminoacids per liter of distilled water) at 220 rpm and 28°C. Total DNA was obtained using the PureLink™ genomic DNA mini kit according to the manufacturer instructions (Invitrogen, Carlsbad, CA, USA). The DNA quality was checked in 0.8% agarose gel electrophoresis, and it was quantified using a NanoDrop spectophotometer ND1000 (Nanodrop Technologies, Wilmington, DE, USA).

### Genotyping with AFLPs

Two hundred nanograms of total DNA from each isolate were digested with the restriction enzymes *Eco*RI and *Mse*I to generate the AFLPs [34], using the AFLPs Core Kit for microorganisms from Invitrogen Corporation, as recommended by the manufacturer (Invitrogen, CA, USA). The following modifications were implemented for the current study: each restricted product was diluted 1:10 and used as template for non-selective PCR amplification with primers *Mse*I + 0/*Eco*RI + 0. The thermal profile used was: 20 cycles at 94°C for 30 sec; 56°C for 60 sec; 72°C for 60 sec. A 1:25 dilution of the PCR product was used as template for the selective amplification with four primer combinations (*Eco*RI + T/*Mse*I + T, *Eco*RI + T/*Mse*I + A *Eco*RI + G/*Mse*I + A and *Eco*RI + C/*Mse*I + A) (Additional file 1). The thermal profile for the selective amplifications was: one cycle at 94°C for 30 sec; 65°C for 30 sec; and 72°C for 60 sec, and 12 cycles with a touch-down in the annealing temperature of 0.7°C per cycle. Finally, 23 cycles were conducted at 94°C for 30 sec, 56°C for 30 sec and 72°C for 60 sec. Selectively amplified products were separated on 6% polyacrylamide gel (19:1 acrylamide:bisacrylamide; 7.5 M Urea; 1× TBE buffer) at 3000 V, 40 mA for 1 hour and 40 minutes on a vertical polyacrylamide electrophoresis apparatus. Every sample was run twice to verify AFLP reproducibility. AFLP bands were detected with silver staining. Polymorphic bands were then scored as either present (1) or absent (0) on a presence/absence matrix. Only strong bands were included in the matrix.

### Selection and evaluation of VNTRs

VNTR loci were selected according to the Hunter-Gaston discriminatory index (HGDI) [35], which was previously evaluated among 65 genomes of *Xam*[36]. Loci with HGDI scores higher than 0.6, such as, XaG1_02, XaG1_29, XaG2_52, XaG1_67 and XaG1_73 were selected to be amplified from *Xam* isolates. The primers used for PCR amplification were those reported by Arrieta et al., [36].

VNTR loci were amplified either from genomic DNA or from a fresh bacterial colony. Each PCR reaction contained 10-50 ng of genomic DNA, 2.5 mM MgCl_2,_ 3 mM PCR primers, 1.3 mM dNTPs and 1 unit of Taq DNA polymerase (Fermentas, USA). Thermal profile was conducted as follows: 3 min at 95°C, 35 cycles consisting of 20 sec at 95°C, 30 sec at 52–58°C, and 60 sec at 72°C, with a final extension step at 72°C for 10 min. When a bacterial colony was used as the direct source of the template, an additional step of 95°C for 10 min at the beginning of the thermal profile was added. Amplified products were separated on 1% agarose gels and then sequenced using the primers listed in the Additional file 1.

Sequences were aligned using MUSCLE [37] and then numbers of complete repeats were calculated from multiple alignments. The number of repeats at each locus for every strain was recorded in a matrix.

### Data analysis

Molecular Variance Analysis (AMOVA) was conducted to determine genetic differentiation among sampled provinces estimating the genetic differentiation among population value (Φ_PT_) with 1000 permutations using GenAlEx 6.5 [38]. Then, genetic Euclidean distances among sampled locations were calculated in GenoDive 2.0b20 [39]. To visualize the dissimilarities among the isolates, a Principal Coordinates Analysis (PCoA) was also carried out using GenAlEx 6.5 [38]. Once the dissimilarities among isolates were confirmed, isolates were clustered in an unrooted distance tree with the Neighbor-Joining algorithm in SplitsTree version 4.12.3 [40]. Branch supports were determined running 1000 bootstrap replicates. Then, current isolates were assigned into genetic populations using a clustering algorithm based on Bayesian model in STRUCTURE 2.3.3 [41] without prior population information. Genetic clusters of the isolates were generated with independent allele frequencies and five thousand replicates during burning period and 100.000 Monte Carlo Markov chain (MCMC) iterations. Iterations were performed from 1 to 10 clusters (K) and then the optimal number of clusters was determined according to Evanno et al. [42]. F_ST_ values [43] from the optimal number of clusters were recorded. A Mantel test was performed with 999 permutations using GenAlEx 6.5 [38] to confirm if the clustering pattern was correlated with geographical distances of sampled locations.

Isolates were then classified into haplotypes, which were established with an infinite allele model and a threshold of 0 using GenoDive 2.0b20 [39]. The clonal diversity at each location was estimated implementing the corrected Nei and Shannon indices in GenoDive 2.0b20. Assigned haplotypes were split in a Minimum Spanning Network using BioNumerics software (version 7.1) created by Applied Maths NV (Available from http://www.applied-maths.com).

## Results

### A large number of isolates was obtained from cassava producing areas in the Eastern Plains of Colombia

A total of 101 isolates were collected at four locations in the Eastern Plains of Colombia. From these, 47 isolates were collected in La Libertad (Meta) from an experimental field that contained 96 representative cassava accessions from the Eastern Plains. The experimental field was visited with permission of the International Center for Tropical Agriculture (CIAT). In contrast, other sampled locations presented one or a maximum of two cassava varieties per field. Commercial field crops at Granada and Fuente de Oro (Meta) presented a comparatively low number of samples with typical CBB symptoms. Only three isolates were obtained from Granada and one isolate was obtained from Fuente de Oro. In addition, 50 *Xam* isolates were obtained from four fields located in Orocué in the province of Casanare. Samples collected in Orocué came from small plots where cassava is cultivated for self-consumption of smallholder farmers, in contrast to the fields visited in the other locations.

### AFLP and VNTR markers showed reproducible band patterns

One-hundred and one isolates and ten reference strains were characterized by both AFLP and VNTR markers. The characterization with AFLPs was performed with four combinations of selective primer pairs. AFLP band patterns obtained with selective amplifications were clear to read after detection with silver staining. A total of 57 polymorphic bands were generated when primer combinations *Eco*RI + T/*Mse*I + T, *Eco*RI + T/*Mse*I + A and *Eco*RI + C/*Mse*I + A were used. Primer combination *Eco*RI + G/*Mse*I + A did not produce polymorphic bands among the evaluated isolates. AFLP selective amplifications were run twice for each isolate. Band patterns were consistent between replicates.

*Xam* isolates were also characterized using five VNTR loci. PCR amplicons of VNTRs were strong and highly reproducible. Sequencing of VNTR loci showed that the number of alleles per locus ranged from 7 to 17 (Table 1). Locus G1_29 was the most polymorphic VNTR with 17 different alleles, ranging from 1 to 23 repeats (Table 1). The data sets supporting the results of this article are available in the GenBank database (Accession numbers XaG1_02: KJ736838 - KJ736944; XaG1_29: KJ736945 - KJ737053; XaG2_52: KJ737163 - KJ737268; XaG1_67: KJ737269 - KJ737369; XaG1_73: KJ737054 - KJ737162) and in the Dryad Digital Repository: http://doi.org/10.5061/dryad.t173v.

**Table 1:** **Characteristics of VNTR loci evaluated in *Xam *** **isolates from the Colombian Eastern Plains**

**VNTR locus**	**Repeat**	**Number of different alleles**	**Range of allele repetitions**	**Dominant alleles**	**HGDI index**
G1_02	TCCCCAT	7	1 - 9	4 8	0.7019
G1_29	ATCCCGA	17	1 - 23	5	0.858
G1_52	CCGCCACAACGCA	7	4 - 10	6	0.5873
G1_67	CGACAC	14	10 - 26	16 26	0.8428
G1_73	GGTCAT	8	5 - 12	6 7 9	0.797

VNTR loci were selected according to discriminant index reported by Arrieta and collaborators [36].

### *Xam* populations presented a genetic differentiation among locations in the Eastern Plains

**Figure 2:**
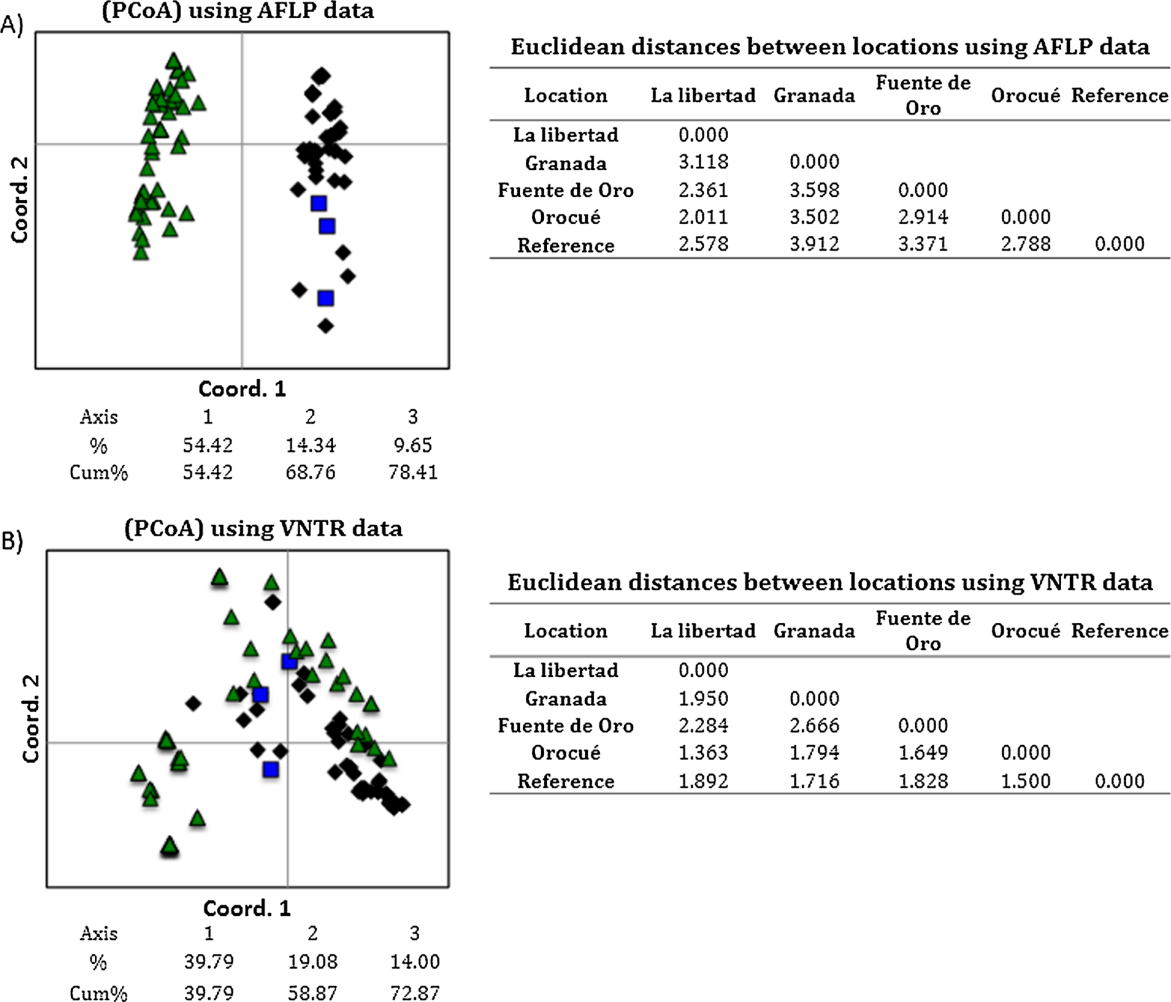
**Discrimination of sampled locations in the Colombian Eastern Plains by AFLP and VNTR markers.** Disimilarities among *Xam* isolates were calculated by a Principal Coordinates Analysis (PCoA). Isolates are represented in the PCoA according to their geographical origin. Triangle: La Libertad; square: Granada; rhombus: Orocué. In addition, genetic distances among sampled locations were calculated using the Euclidean distance. **A)** PCoA was estimated using AFLP data. **B)** PCoA and was estimated using VNTR data. Fuente de Oro was excluded of the PCoA because this location only presented one isolate.

In order to confirm if there was genetic differentiation among sampled locations, an AMOVA was conducted. Φ_PT_ values showed a statistically significant genetic differentiation between each pair of locations (Table 2). The differentiation was evidenced using both types of molecular markers. Similar proportions of genetic variation were obtained when comparisons between locations and within locations were performed using AFLPs. However, 80% of the genetic variation was distributed within the sampled locations when isolates were characterized by VNTRs. Furthermore, PCoA analysis showed that AFLPs allowed the detection of a more contrasting differentiation among isolates with different geographical origins (Figure 2). VNTRs also permitted an evident differentiation, but a partial overlapping of isolates from La Libertad and Orocué was observed. However, approximately 75% of the variation among isolates was explained with the first three coordinates of the analysis for both markers (Figure 2).

**Table 2:** Genetic variance among sampled locations in the Eastern Plains using AFLP and VNTR markers

**Location pair**		**Number of isolates**		**Molecular marker**					
				**AFLP**			**VNTR**		
**Loc. 1**	**Loc. 2**	**Loc. 1**	**Loc. 2**	**Φ**_**PT**_	**LinΦ**_**PT**_	**p-value**	**Φ**_**PT**_	**LinΦ**_**PT**_	**p-value**
La Libertad	Granada	47	3	0.393	0.649	0.001*	0.245	0.324	0.003*
La Libertad	Orocué	47	50	0.520	1.082	0.001*	0.192	0.238	0.001*
Granada	Orocué	3	50	0.623	1.649	0.001*	0.196	0.244	0.021*

* Statistically significant (p > 0.05).

(Φ_PT_): genetic differentiation among population.

(LinΦ_PT_): Linearized genetic differentiation among population.

### The genetic population structure of *Xam* was correlated with the geographical origin of isolates in the Eastern Plains of Colombia

**Figure 3:**
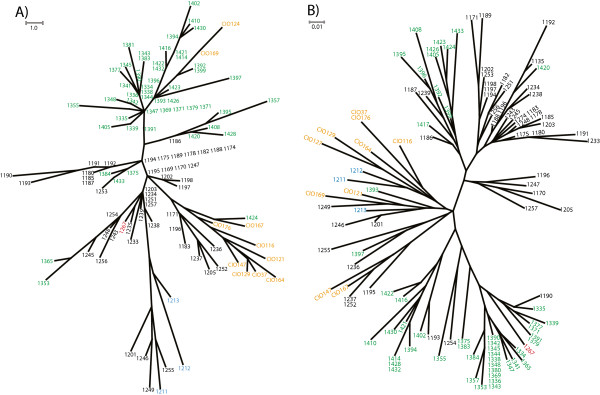
**Distance trees generated with AFLP and VNTR data from isolates collected in Casanare and Meta.** Unrooted distance trees were constructed with the Neighbor-Joining algorithm in SplitsTree version 4.12.3 **A)** Distance tree was constructed using four selective pairs of primers to amplify AFLP markers. **B)** Distance tree constructed using five VNTR loci. La Libertad: black; Granada: blue; Fuente de Oro: red; Orocué: green and reference strains: orange.

Distance trees were constructed using AFLP and VNTR data to determine how genetic distances were distributed among current isolates and reference strains (Figure 3). Tree topologies showed a generalized clustering according to geographical origin of the isolates, but the composition of inner clusters changed between techniques. In most of the cases, the global behavior of isolates across the topologies was comparable, with only few exceptions. One of them was a small group of isolates from Orocué, which clustered together with isolates from La Libertad (Meta) when VNTRs were used. This grouping was not observed when AFLPs were used. Interestingly, both techniques revealed that most of the reference strains tended to cluster with isolates from Orocué (Casanare) and La Libertad (Meta), which suggested that those strains presented a similar proportion of shared characters with strains coming from these two locations. This is supported by the fact that similar Euclidean distances were obtained when reference strains were compared to the isolates from La Libertad and to the isolates from Orocué (data not shown).

**Figure 4:**
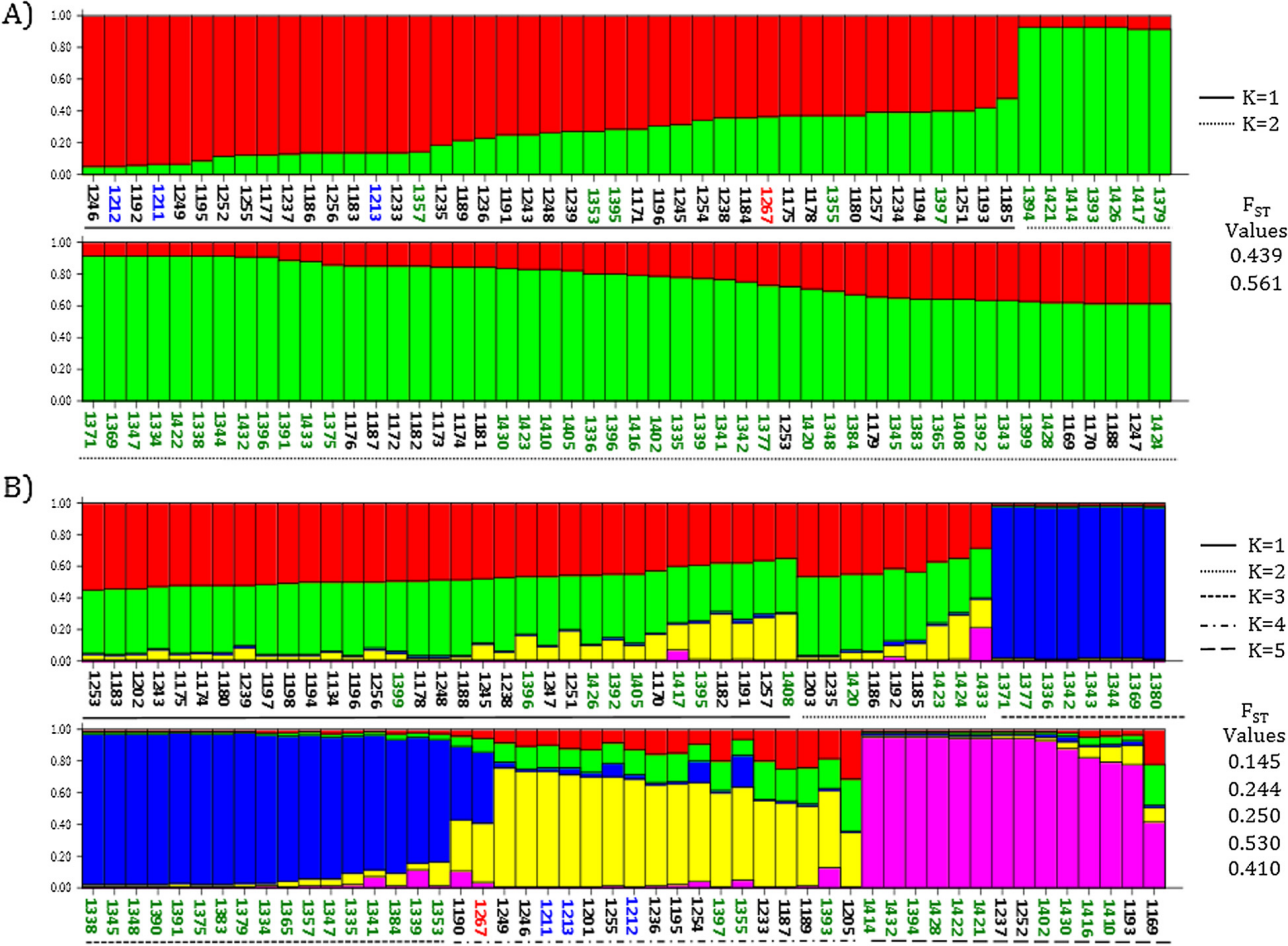
**Estimation of genetic populations of *Xam *in the Eastern Plains using AFLP and VNTR markers.***Xam* isolates were assigned to the optimal number of clusters (K) estimated using STRUCTURE 2.3.3. **A)** Two genetic clusters estimated using AFLP data. **B)** Five genetic clusters estimated among isolates using VNTR data. Each isolate is represented by a single vertical line broken into K-colored segments. Color length in vertical lines represents the proportion of each inferred K clusters for each isolate. Color code of isolates labels represent the geographical origin of isolate: La Libertad: black; Granada: blue; Fuente de Oro: red and Orocué: green. Lines at the bottom delimit each estimated genetic population (K). Fixation index (F_ST_) is indicated for each population.

We then evaluated if there were distinguishable genetic clusters of the pathogen in the Eastern Plains region. When isolates were assigned to estimate genetic clusters using AFLP markers, they were grouped in two well-differentiated genetic clusters (Figure 4A). Each genetic cluster was mainly conformed by isolates from the same location, suggesting that geographical distances influenced the designation of clusters. This observation was corroborated with a Mantel test that showed a positive correlation between genetic and geographical distances (R^2^ = 0.9302). On the other hand, five genetic clusters were estimated when isolates were characterized using VNTRs (Figure 4B). In the same way, K clusters grouped according to the origin of isolates but this was less evident than for the clusters generated by AFLPs. The fact that VNTRs detected new clusters is suggesting that those markers were able to distinguish an encrypted population structure that was not detected by AFLPs. Similarly to what was observed with AFLPs, VNTRs detected a genetic structure correlated with geographical location. The Mantel test suggested a positive correlation between genetic and geographical distances (R^2^ = 0.2666), however this correlation was not as evident as the one estimated using the AFLP markers. F_ST_ values from the populations estimated using both techniques were compared. F_ST_ values of the five populations obtained for the VNTR analysis were lower than the F_ST_ values from the populations generated with the AFLP analysis, indicating that VNTRs detected a higher genetic flow between populations.

### The diversity of *Xam* haplotypes in the Eastern Plains was comparable when the two types of molecular markers were implemented

An analysis of haplotype assignment was conducted to determine the number and distribution of haplotypes among sampled locations. A haplotype was defined with a 100% similarity threshold for both AFLP and VNTR loci. Both approaches generated a highly similar number of haplotypes for each sampled location and for reference strains (Table 3). In addition, both techniques allowed the distinction of a high number of haplotypes, with AFLPs and VNTRs detecting 86 and 87 haplotypes out of 111 isolates, respectively. Consequently, the clonal diversity at each location was considerably high and comparable for both approaches (Table 3). However, high diversity values were most probably the result of the stringency in the assignment of haplotypes (100% similarity between isolates).

**Table 3:** Assignment of haplotypes and clonal diversity in the Colombian Eastern Plains

**Molecular marker**	**Location**	**No. isolates**	**No. haplotypes**	**No. repeted haplotypes**	**Corrected Nei’s index**	**Corrected Shannon’s index**
					**Div_obs**	**Div_obs**
**AFLP**	La Libertad	47	33	4	0.967*	1.802*
	Granada	3	3	-	1.000	nan
	Fuente de Oro	1	1	-	nan	nan
	Orocué	50	39	7	1.000	nan
	Reference	10	10	-	0.985	2.001*
	**Overall**	**111**	**86**	**13**	0.991*	2.331*
**VNTR**	La Libertad	47	39	6	0.988*	2.163*
	Granada	3	3	-	1.000	nan
	Fuente de Oro	1	1	-	nan	nan
	Orocué	50	34	6	0.940*	1.783*
	Reference	10	10	-	0.978	1.653*
	**Overall**	**111**	**87**	**12**	0.984*	2.356*

*Statistically significance (p > 0.05).

nan: non calculated value because all isolates present a different haplotype.

**Figure 5:**
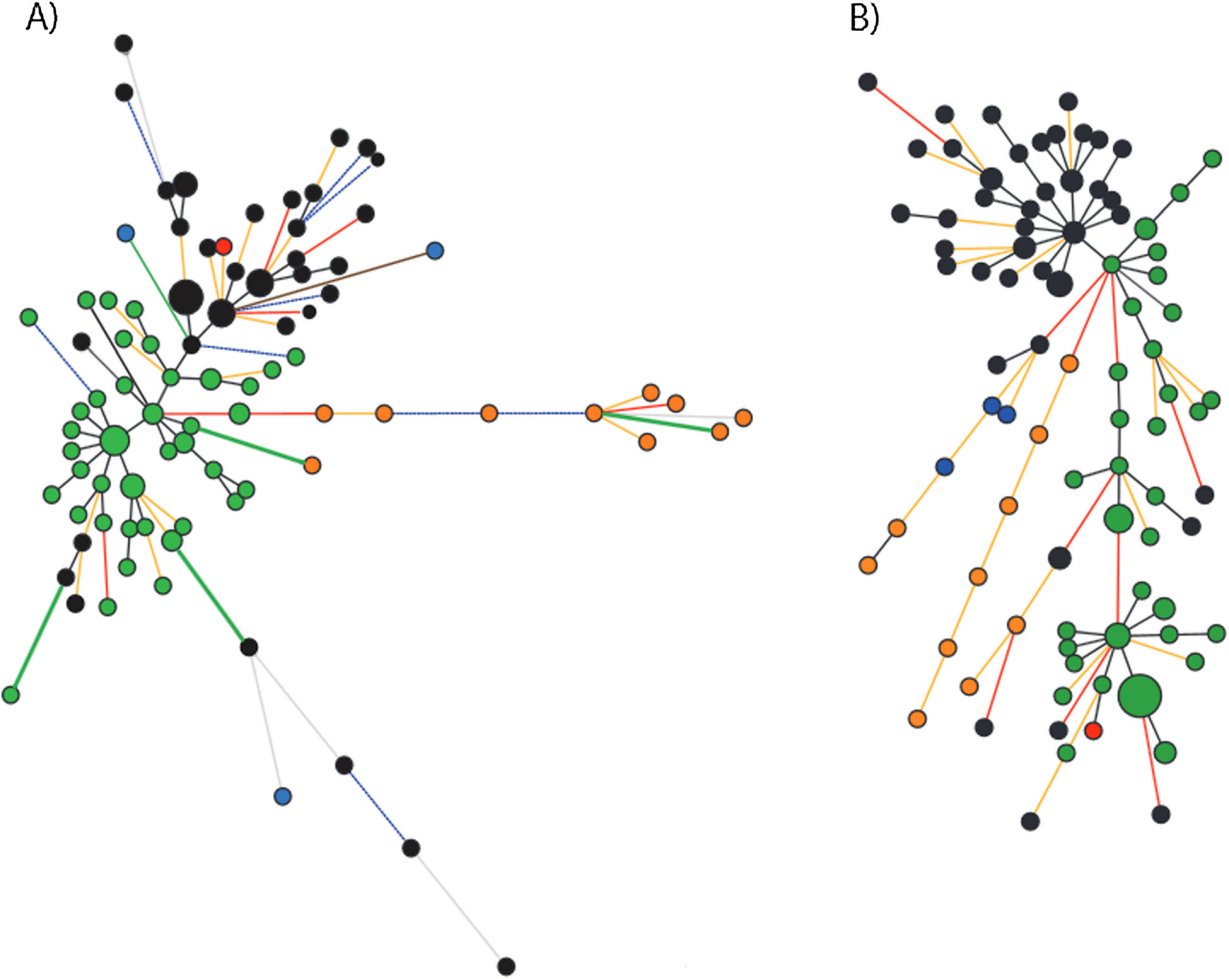
**Connectivity of haplotypes assigned among *Xam *isolates from the Eastern Plains. A)** Haplotype network generated using AFLP data. **B)** Haplotype network generated using VNTR data. Sizes of circles represent the number of isolates belonging to each haplotype. Colors of circles represent the geographical origin of each haplotype. La Libertad: black; Granada: blue; Fuente de Oro: red; Orocué: green and reference strains: orange. Colors of branches represent the number of changes between haplotypes. 1: black; 2: yellow; 3: red; 4: purple; 5: green; 6: gray and 9: brown.

Haplotypes were divided in a minimum spanning network to visualize the connectivity between them (Figure 5). These networks evidenced that most haplotypes are grouped according to geographic location, which was expected from the Mantel test results described above. However, VNTR haplotypes from Orocué (Casanare) presented larger genetic distances among them than to haplotypes from La Libertad (Meta). This result suggests that VNTR amplification was more discriminating for haplotypes contained in the same geographical area. Sometimes, this haplotype discrimination was considerably notorious. For example, haplotypes from the same location, such as Granada (Figure 5), were displayed far from each other in the networks. Finally, it was evident that haplotypes from the reference strains showed a remarkable distance from most of the haplotypes assigned to current *Xam* isolates, evidencing a potential temporal differentiation. This was observed with both types of markers (Figure 5).

## Discussion

In order to determine the current state of populations of *Xam* and the diversity of this pathogen in the Colombian Eastern Plains, *Xam* isolates were characterized using two types of molecular markers. AFLPs were the first molecular markers used for the assessment of diversity in this pathogen and have also been implemented in recent population studies [10,15]. The second type of molecular marker was VNTR, which have recently been proposed as promising markers for typing populations of this pathogen [36] but had not been evaluated for this purpose. Here, we present a complete comparison of population analyses obtained with both types of markers and report the usefulness and benefits of these techniques in the characterization of *Xam* populations.

Sampling for this study was focused on four locations in two provinces of the Eastern Plains of Colombia. Although the sampling effort was equal for each location, it was not possible to obtain comparable amounts of samples from each sampled area. For instance, 96% of the total isolates were collected in La Libertad (Meta) and Orocué (Casanare). In contrast, Fuente de Oro and Granada were the source of only a few samples for this study. The difference in the number of isolates was due to great differences in disease incidence among locations. In contrast to La Libertad and Orocué, cassava fields in Granada and Fuente de Oro are constantly rotated by growers or substituted by other types of crops and this could have contributed to a reduction in the incidence of CBB in these locations.

In spite of the difference in the number of samples, we could easily conclude that VNTRs and AFLPs provide congruent results for *Xam* populations. This is supported by several observations. First, both techniques were able to genetically differentiate the populations of *Xam* between sampled locations. Second, global clustering patterns were constant in both types of markers. For instance, clustering in distance trees and haplotype networks was clearly defined by the geographical origin of isolates, although AFLPs displayed a better geographical clustering (Figure 3). Third, the distribution of haplotypes from Granada (Meta) was congruent between both techniques used. Both of them displayed Granada haplotypes very distant as shown in the Figure 5. This behavior is in contrast to what was expected. Cultural practices such as crop rotation, which is intensively implemented in this location, should have generated a genetic drift event that could have led to a reduction in pathogen diversity [3]. However, the instability of cassava fields due to intensive crop rotation and the reduced number of plants with CBB symptoms in Granada did not allow the constant tracing of the pathogen in order to explain the attained behavior of these isolates. Fourth, a congruent behavior was also observed for the reference strains, which were almost completely grouped in the distance trees and networks from both analyses (Figures 3, 4 and 5). This suggests a temporal differentiation of *Xam* populations, a process that is occurring even in short periods of time, as was evidenced in the recently characterized Caribbean populations and also with populations from the 1990s [9,16].

There were also contrasting results when analyses from AFLPs and VNTRs were compared. For example, although isolates were clustered according to their geographical origin, the composition of inner clusters changed between techniques. This discrepancy could be explained by the fact that each type of marker evaluates polymorphisms at different scales. AFLPs evaluate differences distributed along the whole genome and those differences must be located in recognition sites for restriction enzymes [34]. Detection of polymorphisms in AFLPs is highly influenced by the combination of restriction enzymes and selective primers used in this technique [44]. In contrast, VNTRs evaluate the variation in restricted genomic areas, where short tandem repeats are located. These repetitive genomic regions promote the Slipped-strand mispairing phenomenon during DNA replication, producing a change in the number of repetitive elements and increasing the mutation rate in a specific locus [21,45,46]. In addition, VNTRs could present homoplasy events that could be influencing the clustering process. However, the use of reasonable number of VNTR loci reduces this effect [47]. Because both AFLPs and VNTRs are evolving at different rates and each detects variation at different genomic scales, it is not surprising that the pattern of composition of the inner clusters could differ, as observed at the Figure 3.

Additionally, AFLPs and VNTRs showed discrepancies when the optimal number of genetic clusters was estimated. The optimal K clusters for VNTRs (k = 5) was larger than that for AFLPs (k = 2). This finding suggests that VNTRs were able to detect a more detailed structuring of *Xam* population that was not detected by AFLPs. However, three of the genetic clusters generated by VNTRs presented considerably lower F_ST_ indices indicating a high genetic flow among them (Figure 4). These genetic clusters with a high genetic flow could be considered as part of a bigger population when the other molecular marker is implemented. In our case, STRUCTURE could assume that those three genetic clusters with high genetic flow could be encrypted when the clusters were estimated using AFLP markers. On the other hand, although K clusters presented considerable differences in F_ST_ values, both techniques confirmed the genetic flow between geographically distant locations, such as La Libertad and Orocué, which are separated by approximately 250 km. This process of genetic flow was also documented between distant locations even when locations were located in very distant regions of Colombia. For example, between the Caribbean and the Eastern Plains regions, there is a geographic distance of more than 500 km [8,14,15].

If we compare the current populations from the Caribbean and the Eastern Plains, it is evident that the pathogen is more diverse in the Caribbean. A total of 57 AFLP haplotypes were detected among 160 isolates from the Caribbean region, when using 80% similarity as a threshold. [15]. In the Eastern Plains region, 28 haplotypes were detected among 111 isolates, with haplotype assignment at 80% similarity (data not shown). These observations are in contrast to what was reported for Colombian populations in the nineties, where the pathogen was more diverse in the Eastern Plains than in the Caribbean region [8,9,14]. This could be related to the limited number of samples collected in the Eastern Plains because of the low CBB incidence encountered in some of the sampled locations at this region. The decrease in incidence could be explained by the reduction in the area dedicated to cassava cultivation in Meta in recent years [48].

In contrast to the locations at the Eastern Plains, most of the Caribbean populations did not display a geographically-dependent genetic differentiation [15]. These differences could be a consequence of the mode of cultivation of cassava in the two regions. Cassava cropping in the Caribbean is considerably more intensive and extensive than it is in the Eastern Plains [48], something that could reduce geographical isolation of *Xam* populations. In contrast, the geographical differentiation detected at the Eastern Plains populations could also be associated with the fact that growers in Orocué are indigenous people who do not move over large geographical distances. This phenomenon could reduce the exchange of propagative material infected with *Xam,* hence enhancing genetic differentiation between Eastern Plain locations.

In this study, we were able to assess the usefulness of VNTRs for the study of *Xam* populations. Remarkably, only 5 VNTR loci offered a very similar panorama of the pathogen populations to that obtained by 57 AFLP loci. This finding is relevant for further studies on the population dynamics of *Xam*, because VNTR markers provide a faster and less expensive characterization of bacterial isolates, as has been reported for several pathogenic microorganisms [22,24,25,49]. The fact that amplification of VNTRs requires neither a complex DNA extraction procedure, nor compounds different from those used in a regular PCR, makes VNTRs ideal when a large number of isolates are considered and when funding is limiting. Moreover, sharing information between laboratories would be considerably more straightforward with VNTRs than with AFLPs, because results from VNTRs can be more easily coded [17]. For future *Xam* survey studies we recommend the use of VNTRs. The rising number of sequenced genomes available nowadays, provides an additional advantage to identify new VNTR loci, hence improving the characterization of several pathogens [19,21,50,51]. Recently, 65 partial genomes of *Xam* strains have been released [52], providing a valuable opportunity to detect VNTRs with high discriminatory power. Currently, we are focusing on the prediction and evaluation of new VNTR loci into a core of the representative *Xam* strains using the information obtained from the 65 draft genome sequences. Our goal is to obtain a small sets of VNTRs with a high discriminatory power, aiming to implement them in studies that involve a large number of isolates to provide a more accurate description of evolving processes taking place in *Xam* populations.

## Conclusions

This study represents the first attempt to type populations of *Xam* using VNTRs as molecular markers. Here we demonstrated that a small number of VNTR loci could offer a similar panorama of the status of the pathogen to that offered by AFLPs markers. Because VNTRs represent a fast and simple tool to type *Xam* populations, their implementation will allow a constant and adequate surveillance of the pathogen, which could provide information to improve the efficiency of strategies for disease control, such as the deployment of resistant varieties.

### Availability of supporting data

The data sets supporting the results of this article are available in the Dryad Digital Repository: http://doi.org/10.5061/dryad.t173v.

DNA sequences are available in Genbank database: (Accession numbers XaG1_02: KJ736838 - KJ736944; XaG1_29: KJ736945 - KJ737053; XaG2_52: KJ737163 - KJ737268; XaG1_67: KJ737269 - KJ737369; XaG1_73: KJ737054 - KJ737162).

### Ethics statement

This study did not involve any human material, or human data. No experimental procedure was performed in vertebrate or invertebrate animals for the development of this research.

## Competing interests

The authors declare that they have no competing interests.

## Authors’ contributions

CT was involved in the conception and design of the study, sampling, bacterial isolation, molecular characterization using AFLPs and VNTRs, data analyses and who wrote the manuscript. NAR performed DNA extraction, the evaluation of 3 VNTR loci, VNTR data analyses and drafting of the manuscript. LP contributed in the evaluation 3 VNTR loci, VNTR data analyses and drafting of the manuscript. CM carried the sampling and data acquisition. AT participated in the data acquisition and revised the content of the manuscript. SR was involved in the conception and design of the study, drafting and revising the manuscript. RK was involved in the conception and design of the study and the design of the VNTR strategy. AB participated in the conception and design of the project, funding acquisition, editing and revisiting of manuscript. All authors read and approved the final manuscript.

## Supplementary Material

Additional file 1 Primers used for the AFLP amplification and VNTR amplification and sequencing. Click here for file
