# Supplementary material for: Population typing of the causal agent of cassava bacterial blight in the Eastern Plains of Colombia using two types of molecular markers
Source: BMC Microbiol. 2014 Jun 19;14:161. doi: 10.1186/1471-2180-14-161 (PMC4071214; doi:10.1186/1471-2180-14-161)
Supplement: Additional file 1 — Primers used for the AFLP amplification and VNTR amplification and sequencing. [file 1471-2180-14-161-S1.pdf]

Additional File 1. Primers used for the AFLP amplification and VNTR amplification and sequencing.

Primer Name	Primer sequence
TALE1$_{XAM}$ Fw	tctagatttgatgaagccatgacg
TALE1$_{XAM}$ Rv	aagcttcatcttgttcccacatcacg
EcoRI+0	gactgcgtaccaattc
MseI+0	gatgagtcctgagtaa
EcoRI+T	gactgcgtaccaattct
EcoRI+G	gactgcgtaccaattcg
EcoRI+C	gactgcgtaccaattcc
MseI+T	gatgagtcctgagtaat
MseI+A	gatgagtcctgagtaaa
XaG1_02.1	gctggtgcgcatgctgac
XaG1_02.2	ttcgcggaaggtcatggg
XaG1_29.1	ctacgaggcacctgcgca
XaG1_29.2	catggcatcgcgctgaag
XaG2_52.1	caaaagcccgcagcgatg
XaG2_52.2	ggcgatttcgggctgagg
XaG1_67.1	gtcggcgcgatcgtcatg
XaG1_67.2	acgcgttgtcgctgggc
XaG1_73.2	cacgcccaagcccatcca
XaG1_73.2	caccgaggtcgcggcatc
